# Clinical and prognostic significance of aberrant T-cell marker expression in 225 cases of *de novo* diffuse large B-cell lymphoma and 276 cases of other B-cell lymphomas

**DOI:** 10.18632/oncotarget.16532

**Published:** 2017-03-23

**Authors:** Naoko Tsuyama, Daisuke Ennishi, Masahiro Yokoyama, Satoko Baba, Reimi Asaka, Yuko Mishima, Yasuhito Terui, Kiyohiko Hatake, Kengo Takeuchi

**Affiliations:** ^1^ Division of Pathology, The Cancer Institute, Japanese Foundation for Cancer Research, Tokyo, Japan; ^2^ Department of Hematology, Oncology and Respiratory Medicine, Okayama University Graduate School of Medicine, Dentistry and Pharmaceutical Sciences, Okayama, Japan; ^3^ Department of Hematology Oncology, The Cancer Institute Hospital, Japanese Foundation for Cancer Research, Tokyo, Japan; ^4^ Pathology Project for Molecular Targets, The Cancer Institute, Japanese Foundation for Cancer Research, Tokyo, Japan

**Keywords:** diffuse large B-cell lymphoma, CD5, CD8, aberrant expression, T-cell marker

## Abstract

Expression of T-cell markers, generally investigated for immunophenotyping of T-cell lymphomas, is also observed in several types of B-cell lymphomas, including diffuse large B-cell lymphoma (DLBCL). We previously reported that CD5 expression in DLBCL is an inferior prognostic factor in the era of rituximab. However, data regarding the frequencies, histological relevance, and prognostic importance of T-cell markers other than CD5 are currently unavailable. In the present study, we comprehensively evaluated the expression of T-cell markers (CD2, CD3, CD4, CD5, CD7, and CD8) in 501 B-cell lymphomas, including 225 DLBCLs, by flow cytometry and subsequent immunohistochemistry. T-cell markers other than CD5, such as CD2, CD4, CD7, and CD8, were expressed in 27 (5%) patients, and notably, all of these cases were classified as large B-cell lymphoma subtypes: 25 DLBCLs and 2 intravascular large B-cell lymphomas. CD5 and other T-cell markers were expressed in 15% (31/225) and 10% (25/225) of DLBCL cases, respectively. Five of them co-expressed CD5 and other T-cell markers. Retrospectively analyzing the prognostic relevance of T-cell markers in 169 patients with primary DLBCL treated with rituximab-based chemotherapy, we showed that only CD5 was a strong predictor of poor survival. This study provides information about the occurrence of T-cell markers other than CD5 in B-cell lymphomas, their frequent histological subtypes, and their prognostic significance in DLBCL. CD5 was reconfirmed as a negative prognostic marker in DLBCL patients receiving rituximab-inclusive chemotherapy, whereas T-cell markers other than CD5 were found to have no impact on clinicopathological and survival analyses.

## INTRODUCTION

Lymphocytes are generally subdivided into B, T, and natural killer cells, depending on their lineage markers: CD19, CD20, CD22, and immunoglobulins for B cells; CD2, CD3, CD4, CD5, CD7, and CD8 for T cells; and CD56 for natural killer cells. Lymphoma cells, which are the neoplastic counterparts of lymphocytes, usually retain the lineage marker expression pattern of their normal counterparts. Therefore, lymphomas are subclassified according to the expression pattern of their markers. However, in some lymphomas, aberrant expression of lineage markers is observed.

CD5 is primarily expressed by T cells, but is also expressed by a small subset of B cells [1]. In B-cell neoplasms, CD5 is usually expressed in chronic/small lymphocytic lymphomas (CLLs/SLLs) and mantle cell lymphomas (MCLs), for which the normal counterparts are considered to be CD5-positive B cells. However, other B-cell lymphomas may sometimes express CD5 aberrantly. Approximately 10% of the diffuse large B-cell lymphomas (DLBCLs) express CD5 [2, 3], and such cases comprise an immunohistochemical subgroup of DLBCL, according to the 2008 WHO classification [4]. Therefore, CD5-positive DLBCL has been well studied, and some researchers claim that it has a worse prognosis than CD5-negative DLBCL [2, 3].

In contrast to CD5, the significance of other T-cell markers rarely expressed in B-cell lymphomas remains unclear. Although B-cell lymphomas with T-cell markers other than CD5, such as CD8^+^ CLL [5–13] or CD2/CD3/CD4/CD7/CD8^+^ large B-cell lymphoma [13–31] have been reported (Table 1), data regarding their frequency, histopathological distribution, and clinical features are rarely available, because the number of identified cases in each report is small. This rarity may partly be attributed to the differences in the immunophenotypic procedures used. In immunohistochemistry (IHC), a limited number of lineage markers, such as CD3, CD20, and/or other frequently expressed lineage markers, are usually examined first; other less frequently expressed markers are subsequently investigated for further subclassification. This convention means that once a case is determined to be of B-cell lineage, it may be further examined for other less frequently expressed B-cell markers, but not T-cell markers.

**Table 1:** Non-CD5-T-cell marker-positive large B-cell lymphomas: literature review

References	Total no. of cases	No. of positive cases and subtype	T-cell marker (no. of positive/tested cases)						B-cell marker (no. of positive/tested cases)			Gene rearrangement		EBER-ISH	Detection method
			CD2	CD3	CD4	CD7	CD8	CD5	CD20	PAX5	CD138	IGH/K	TCRB/G		
Suzuki *et al*.	150	11 DLBCL	4/11	0/11	1/11	6/11	2/11	1/11	10/11	NA	NA	NA	NA	1/11	FCM with IHC
Lee *et al*.	1	1 DLBCL-E	0	1	0	NA	0	0	1	1	NA	1	1	1	IHC only
		2 PBL	0/2	2/2	1/2	0/2	0/2	0/2	1/2	1/2	2/2	1/1	0/1	2/2	IHC only
Oliveira *et al*.	16	7 DLBCL-PCD	0/7	7/7	2/7	0/7	0/7	0/7	7/7	7/7	2/2	7/7	0/7	1/7	IHC only
		5 DLBCL-AF	5/5	5/5	4/5	0/5	2/5	4/5	2/5	5/5	NA	4/4	0/4	0/4	
		2 BL	0/2	2/2	0/1	0/1	0/1	0/2	2/2	1/1	NA	0/0	0/0	0/2	
Sun *et al*.	1	1 PBL	0	1	NA	0	NA	0	0	0	1	NP	NP	1	IHC only
Sangle *et al*.	1	1 DLBCL	1	0	0	1	0	0	1	1	NA	0	0	0	IHC with FCM*
Wang *et al*.	1	1 PMLBL	0	1	0	0	0	0	1	1	0	1	0	0	IHC only
Suzuki *et al*.	1	1 PBL	NA	1	1	NA	NA	NA	0	0	1	1	0	1	IHC only
Wang *et al*.	4	2 DLBCL	1/2	2/2	1/2	2/2	0/2	1/2	2/2	2/2	0/2	2/2	0/2	2/2	IHC only
		2 PBL	0/2	2/2	0/2	0/2	0/2	0/2	0/2	0/2	2/2	2/2	0/2	1/2	
Laurent *et al*.	27	11 ALK+ DLBCL	0/11	0/11	11/11	NP	0/11	0/11	1/11	NA	11/11	1/2	NA	NA	IHC only
Carulli *et al*.	951	2 DLBCL	NA	NA	NA	NA	2/2	NA	NA	NP	NP	NP	NP	NP	FCM only
Tzankov *et al*.	1	1 PBL	NA	0	1	NA	NA	NA	NA	NA	1	1	1	1	IHC only
Tomita *et al*.	1	1 PAL	0	0	0	1	0	0	0	NA	NA	1	0	1	IHC and FCM
Petitjean *et al*.	12	5 PAL	4/5	4/5	1/5	0/5	0/5	0/5	3/5	NP	1/4	1/1	0/1	5/5	IHC only
Kaleem *et al*.	210	2 DLBCL	1/2	0/2	1/2	1/2	0/2	0/2	NA	NP	NP	NP	NP	NP	FCM only
Inaba *et al*.	128	1 DL	1	0	0	3	0	0	3/4	NP	NP	NDD	NDD	NP	FCM only
Beaty *et al*.	1	1 HHV+ DLBCL	NP	1	NP	NP	NP	NP	1	NP	NP	NP	NP	1	IHC only
Mori *et al*.	1	1 PAL	0	1	1	NP	0	0	1	NP	NP	0	0	1	IHC only
Hollingsworth *et al*.	3	1 IDA lymphoma	0	1†	0	0	0	0	0	NP	NP	1	1	1	FCM and/or IHC
Present series	501	25 DLBCL‡	2/25	0/25	0/25	10/25	18/25	5/25	25/25	NP	NP	11/11	0/9	0/25	FCM with IHC
		2 IVLBCL	0/2	0/2	1/2	0/2	1/2	2/2	2/2	NP	NP	1/1	0/1	0/2	

^*^ The details of the flow cytometry result are not available; †positive by immunohistochemistry and negative by flow cytometry; ‡Cases include one patient with follicular lymphoma grade 3B and diffuse large B-cell lymphoma (DLBCL); IGH/K: immunoglobulin heavy chain/kappa light chain; TCRB/G, T-cell receptor beta chain/gamma chain; NA, not available; DLBCL-E, Epstein-Barr virus positive DLBCL of the elderly; DLBCL-PCD, DLBCL with plasmacytic differentiation; DLBCL-AF, DLBCL with anaplastic features; NP, not performed; DL, diffuse large cell lymphoma (classification according to Working Formulation); NDD, not described in detail; IDA lymphoma, immunodeficiency associated lymphoma.

In the present study, we investigated the frequency and histopathological distribution of T-cell marker-positive mature B-cell lymphomas in 501 cases, the largest number so far to be consecutively screened by flow cytometry (FCM). Every T-cell marker-positive case was validated by IHC. We identified 27 cases with T-cell markers other than CD5, and investigated their clinicopathological characteristics. Furthermore, to clarify the clinical relevance of T-cell markers other than CD5 in primary DLBCL, we retrospectively analyzed 169 patients with DLBCL, who received rituximab plus cyclophosphamide, doxorubicin, vincristine, and prednisone (R-CHOP)-based chemotherapy, and follow-up at our institution.

## RESULTS

### Frequencies of T-cell marker detections in B-cell lymphomas and correlation with histopathological classification

**Figure 1:**
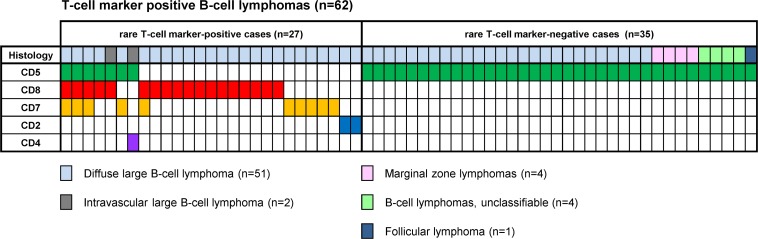
T-cell marker detections in B-cell lymphomas other than CLL/SLL and MCL Distribution of T-cell markers, including CD5 and other T-cell markers, in 62 cases of B-cell lymphomas other than CLL/SLL and MCL.

Of the 501 mature B-cell lymphoma cases analyzed, 92 cases were positive for T-cell marker(s), including DLBCL-not otherwise specified (DLBCL) (51/225, 23%), MCL (17/17, 100%), CLL/SLL (13/13, 100%), marginal zone lymphoma (MZL) (4/81, 5%; 2 extranodal, 2 nodal), intravascular large B-cell lymphoma (IVLBCL) (2/2, 100%), follicular lymphoma (FL) (1/134, 0.7%), and low-grade B-cell lymphoma unclassifiable (4/19, 21%) cases. After excluding patients with CLL/SLL and MCL (because their cases arise from CD5-positive B-cells by definition), among 62 cases positive for T-cell marker(s), the most frequently expressed T-cell marker was CD5 (n = 43), followed by CD8 (n = 18), CD7 (n = 10), CD2 (n = 2), and CD4 (n = 1) (Figure 1). Eight of these 62 cases were found to express more than one T-cell marker. No CD3-positive cases were identified.

T-cell markers other than CD5 (CD8, CD7, CD2, and CD4) were found in 27/501 (5%) cases, all of which were histologically categorized as large B-cell lymphomas: 25 DLBCL, including one case accompanied by FL grade 3B, and 2 IVLBCL cases (Figure 1 and Table 2). These T-cell markers were detected at a significantly higher proportion in DLBCL than in other common types of B-cell lymphomas (DLBCL vs. FL: 25/225 vs. 0/134, *p* < 0.001; DLBCL vs. MZLs: 25/225 vs. 0/81, *p* = 0.001). The frequency of CD5 and other T-cell marker detection in DLBCL was 15% (31/225) and 10% (25/225), respectively. We observed co-expression of CD5 and other T-cell marker(s) in 5/31 CD5-positive DLBCLs (16%).

**Table 2:** Immunohistochemical and molecular findings for 27 patients with non-CD5-T-cell marker-positive B-cell lymphoma

Case No.	Histology	Flow cytometry/Immunohistochemistry*									Cell of origin	Light chain	MYC/BCL2	EBER-ISH	IGH/TCR	FISH split assay		
		CD2	CD3	CD4	CD5	CD7	CD8	CD8β^†^	CD20	CD10						BCL2	BCL6	MYC
1	DLBCL	−	−	−	ED/+	EN/+	ED/+	+	+/+	−/−	GC	K	−/+	−	NA/NA	−	−	−
2	DLBCL	−	−	−	−/−	−	D/+	−	+/+	−/−	non-GC	K	−/+	−	+/−	−	−	−
3	DLBCL	−	−	−	DN/I	−	ED/+	−	+/+	−/−	non-GC	K	−/+	−	NA/NA	−	−	−
4	DLBCL	−	−	−	DN/+	ED/+	ED/+	−	+/+	−/−	non-GC	Λ	+/+	−	NA/NA	−	+	−
5	DLBCL**^§^**	−	−	−	−/−	−	ED/+	−	+/+	−/−	non-GC	K	−/+	−	+/NA	−	−	−
6	DLBCL	−	−	−	−/−	−	D/+	−	+/+	+/+	GC	Λ	−/+	−	NA/NA	−	−	−
7	DLBCL	−	−	−	−/−	−	D/+	−	+/+	D/+	GC	Λ	+/−	−	NA/NA	−	−	−
8	IVLBCL	−	−	−	E/+	−	D/−	−	+/+	−/−	non-GC	K	−/−	−	NA/NA	−	+	NA
9	DLBCL	−	−	−	D/+	DN/+	DN/+	−	+/+	+/+	GC	Λ	−/+	−	NA/NA	−	−	−
10	DLBCL	−	−	−	−/−	−	D/+	−	+/+	+/+	GC	Λ	−/+	−	+/−	+	+	−
11	DLBCL	−	−	−	−/−	−	ED/+	+	+/+	+/+	GC	K	+/+	−	+/−	+	−	+
12	DLBCL	−	−	−	−/−	−	D/+	−	+/+	+/+	GC	Λ	−/+	−	+/−	−	+	−
13	DLBCL	−	−	−	−/−	−	D/+	−	+/+	+/+	GC	K	−/+	−	NA/NA	−	−	−
14	DLBCL	−	−	−	−/−	−	D/+	−	+/+	+/+	GC	K	NA/+	−	NA/NA	−	−	−
15	DLBCL	−	−	−	−/−	−	DN/+	−	+/+	−/−	non-GC	K	−/+	−	+/−	−	−	NA
16	DLBCL	−	−	−	−/−	−	E/+	+	+/+	−/−	non-GC	Λ	+/+	−	+/−	−	−	−
17	DLBCL	−	−	−	−/−	−	E/+	−	+/+	−/−	non-GC	K	−/+	−	NA/NA	−	−	−
18	DLBCL	−	−	−	−/−	E/+	D/−	−	+/+	−/−	non-GC	K	−/+	−	+/NA	−	−	−
19	DLBCL	−	−	−	−/−	D/+	−	NP	+/+	−/−	non-GC	K	+/+	−	NA/NA	−	−	−
20	DLBCL	−	−	−	−/−	E/+	−	NP	+/+	−/−	non-GC	K	+/+	−	NA/NA	−	−	+
21	DLBCL	−	−	−	−/−	DN/+	−	NP	+/+	−/−	non-GC	Λ	−/+	−	+/−	−	+	−
22	DLBCL	−	−	−	−/−	DN/I	−	NP	+/+	+/+	GC	K	+/+	−	+/−	−	+	+
23	DLBCL	−	−	−	−/−	D/+	−	NP	+/+	−/−	non-GC	−^¶^	+/+	−	NA/NA	−	−	−
24	DLBCL	−	−	−	DN/+	D/+	−	NP	+/+	−/−	non-GC	K	−/+	−	NA/NA	NA	NA	−
25	DLBCL	D/I	−	−	−/−	−	−	NP	+/+	−/−	non-GC	Λ	−/−	−	NA/NA	−	−	−
26	DLBCL	D/+	−	−	−/−	−	−	NP	+/+	−/−	GC	Λ	−/−	−	NA/NA	−	−	−
27	IVLBCL	−	−	D/I	+/+	−	−	NP	+/+	−/−	non-GC	Λ	+/+	−	+/−	−	+	−

^§^Case 5 is a patient with DLBCL of the colon associated with an FL grade 3B component in the tonsil. *For T-cell markers other than CD5, the results of immunohistochemistry are given for only flow cytometry positive cases; ^†^only the results of immunohistochemistry are given; ^¶^absence of expression for both kappa and lambda; IGH, immunoglobulin heavy chain gene rearrangement by southern blot analysis; TCR, T-cell receptor β chain gene rearrangement by southern blot analysis; NP, not performed; NA, not available; E, equal; ED, equal to dim; EN, equal to negative; D, dim; DN, dim to negative; I, indeterminate.

Light chain restriction by FCM was observed in 26/27 B-cell lymphomas positive for T-cell markers other than CD5. Clonal immunoglobulin heavy chain (*IgH*) rearrangement was found in all of the tested cases (12/12) using Southern blot analysis. Meanwhile, FCM analysis detected no expression of either surface CD3 or T-cell receptor (TCR) αβ. Southern blot analysis detected no clonal *TCR* gene rearrangement, in all tested cases (Table 2). These findings strongly supported the B-cell nature of the 27 cases positive for T-cell markers other than CD5.

### Clinicopathological findings for patients with T-cell markers other than CD5

The clinical data of 27 patients with expression of T-cell marker(s) other than CD5 are presented in Table 3. The patients included 19 males and 8 females, aged 41– 82 years (median 65.5). Ann Arbor stage III or IV was observed in 14/27 (52%) patients. The majority of the cases had extranodal involvement (24/27, 89%). We compared the baseline clinical characteristics of 223 DLBCL patients stratified based on T-cell marker status (Table 4): T-cell marker-negative DLBCL (n = 175; 78%), CD5-positive DLBCL (n = 31; 14%), and non-CD5-T-cell-marker-positive DLBCL (n = 17; 8%). Compared to T-cell marker-negative DLBCLs, CD5-positive DLBCLs showed a significant difference in female-male ratio (*p* = 0.0059), poor performance status (PS > 1; *p* = 0.0290), extranodal involvement (*p* = 0.0133), and non-germinal center (non-GC) phenotype (*p* = 0.0043). However, between T-cell marker-negative and non-CD5-T-cell marker-positive DLBCLs, no significant differences in any of the clinical parameters were observed.

**Table 3:** Clinical characteristics of 27 patients with non-CD5-T-cell marker-positive B-cell lymphoma

Case No.	Age/sex	Main site(s) of involvement	Stage	Extranodal involvement			Treatment	Response	Follow-up (months)	Status
				≥ 1	≥ 2	BM				
1	64/M	Cecum	IIA	+	−	−	Sur + R-CHOP	CR	68	Alive, CR
2	71/M	oral cavity	IIA	+	−	−	R-CHOP	NA	64	DOD
3	77/M	mediastinum	IVB	+	+	−	R-CHOP	PD	7	NA
4	66/F	axillary LN	IVB	+	+	−	R-CHOP + RT	PD	24	DOD, refractory
5	41/M	tonsil, colon	III	+	+	−	R-CHOP	CR	71	Alive, CR
6	44/F	cervical LN	IB	−	−	−	R-CHOP + RT	CR	53	Alive, CR
7	74/F	oral cavity	I	+	−	−	R-CHOP	CR	65	Alive, CR
8	58/M	BM, spleen	IVB	+	+	+	R-CHOP	CR	15	DOD, relapsed
9	62/F	nasal cavity	IIA	+	−	−	R-CHOP	PR	34	AWD, relapsed
10	58/M	inguinal LN	IVA	+	−	−	R-CHOP	CR	52	Alive, CR
11	75/F	abdominal LN	IIIA	+	+	+	R-CHOP	PD	2	DOD, refractory
12	60/M	maxillary sinus	I	+	−	−	R-CHOP	CR	46	Alive, CR
13	81/M	Orbit	IA	+	−	−	R-CHOP	CR	7	Alive, CR
14	67/M	abdominal LN	IVA	+	+	−	R-ICE	CR	47	Alive, CR
15	62/M	cervical LN	IIIA	−	−	−	R-CHOP	CR	71	AWOD, relapsed
16	60/M	parotid gland	IIA	+	−	−	R-CHOP	CR	17	Alive, CR
17	78/F	abdominal LN	IV	+	−	−	R-CHOP	CR	26	AWD, relapsed
18	82/M	nasal cavity	IA	+	−	−	R-CHOP	CR	45	Alive, CR
19	64/M	Orbit	IB	+	−	−	R-DHAP	PD	2	DOD
20	81/M	soft tissue (leg)	IVA	+	+	−	R-CHOP	CR	40	DOD, relapsed
21	55/M	cervical LN	IVA	+	+	−	R-CHOP	CR	12	DOD, relapsed
22	43/M	Gingiva	IA	+	−	−	R-CHOP	CR	60	Alive, CR
23	78/M	Orbit	NA	+	+	NA	NA	NA	NA	NA
24	65/M	Prostate	IVA	+	+	−	R-CHOP	CR	49	Alive, CR
25	81/F	nasal cavity	IIA	+	+	−	R-CHOP	NA	57	DOD
26	57/M	cervical LN	IA	−	−	−	R-CHOP + RT	CR	87	Alive, CR
27	73/F	BM	IVB	+	+	+	R-CHOP	PD	10	DOD, refractory

M, male; F, female; BM: bone marrow; LN, lymph node; Sur, surgery; RT, radiation therapy; CR, complete response; PD, progressive disease; PR, partial response; NA, not available; DOD, died of disease; AWD, alive with disease; AWOD, alive without disease; R-ICE, rituximab plus ifosfamide, etoposide, and carboplatinum; R-DHAP, rituximab plus dexamethasone, cytarabine, and cisplatinum.

**Table 4:** Baseline clinical characteristics of 223 patients with DLBCL according to T-cell marker status

Characteristics	All DLBCL	T-cell marker-negative	T-cell marker-positive			P†	P‡	P§
			Total	CD5-positive	Non-CD5-positive			
	n = 223	n = 175	n = 48	n = 31	n = 17			
Median age, range (yeas)	66, 24–95	66, 24–95	66, 40–86	66, 40–86	62, 41–81	0.8937	0.7627	0.9606
Age > 60	154 (69%)	123 (70%)	31 (65%)	22 (71%)	9 (53%)	0.4828	0.1717	1.0000
Sex: male	120 (54%)	99 (57%)	21 (44%)	9 (29%)	12 (71%)	0.1414	0.3122	0.0059*
Stage: 3–4	108 (48%)	83 (47%)	25 (52%)	17 (55%)	8 (47%)	0.6261	1.0000	0.5592
Performance status > 1	22 (10%)	15 (9%)	7 (15%)	7 (23%)	0 (0%)	0.2718	0.3696	0.0290*
IPI: 3–5	68 (30%)	52 (30%)	16 (33%)	12 (39%)	4 (24%)	0.7236	0.7817	0.3996
LDH > normal	118 (53%)	91 (52%)	27 (56%)	19 (61%)	8 (47%)	0.6278	0.8014	0.4352
Extranodal involvement > 1	44 (20%)	30 (17%)	14 (29%)	12 (39%)	2 (12%)	0.0689	0.7428	0.0133*
CNS involvement	9 (4%)	6 (3%)	3 (6%)	3 (10%)	0 (0%)	0.4088	1.0000	0.1376
non-GC phenotype	85 (38%)	58 (33%)	27 (56%)	19 (61%)	8 (47%)	0.0044*	0.2883	0.0043*

CNS, central nervous system; non-GC, non-germinal center. †T-cell marker-negative DLBCL vs. T-cell marker-positive DLBCL; ‡T-cell marker-negative DLBCL vs. non-CD5-T-cell marker-positive DLBCL; §T-cell marker-negative DLBCL vs. CD5-positive DLBCL; *statistically significant (p < 0.05).

**Figure 2:**
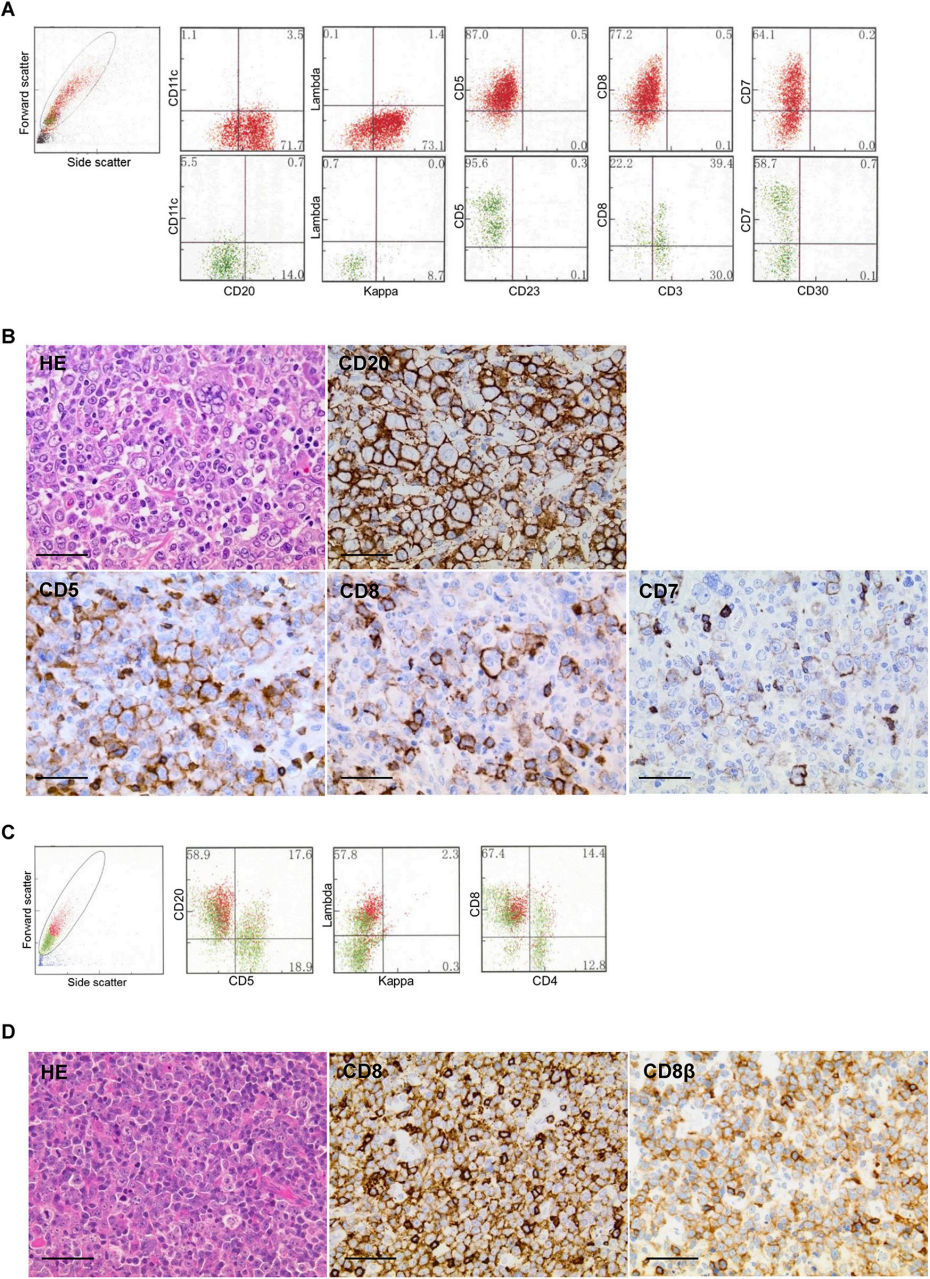
Morphological and immunophenotypic features of T-cell marker-positive diffuse large B-cell lymphomas A and B, case 1; C and D, case 16. Scale bars represent 50 μm. **(A)** Flow cytometry (FCM) showed an abnormal large cell population (red colored plots) positive for CD20, kappa chain, CD5, CD8, and CD7, with a broad range of fluorescent intensity from cells negative or dimly positive for CD7. **(B)** Histologically, lymphoma cells showed a pleomorphic large cell morphology containing multinucleated giant cells (hematoxylin and eosin stain, HE; ×40). Immunohistochemistry showed strongly positive diffuse staining for CD20, weakly positive staining for CD5, and focally positive staining for CD8 and CD7. **(C)** FCM showed the formation of clusters, with positive staining for CD20, lambda chain, and CD8 (red colored plots). The intensity of CD8 was as strong as that of the background normal T cells (green colored plots). **(D)** The cell morphology showed centroblastic large cell infiltrates (HE, ×60). Immunohistochemically, the majority of the lymphoma cells were positive for both CD8 and CD8β. Note that the positive staining of T-cell markers on tumor cells was weaker than on admixed normal T cells, in both case 1 and case 16.

Histologically, all cases with T-cell marker(s) other than CD5 were categorized as large cell lymphomas, and showed atypical large lymphoid cell proliferation with a centroblastic or anaplastic morphology, positive for CD20 (Figure 2). Expression of T-cell markers other than CD5 was successfully detected in 22/27 (81%) cases using IHC. Non-validated cases were dimly positive in FCM analysis and showed negative or indeterminate staining in IHC analysis (Table 2). The available cytogenetic analyses revealed that their karyotypes were complex with numerical abnormalities (Supplementary Table).

Sequential FCM data were available for 7/27 patients. In four of the patients (cases 8, 9, 15, and 27), concordant positive expression of T-cell marker was shown at different sites and at different times of diagnosis, whereas expression in the other 3 patients (cases 11, 17, and 21) exhibited a discrepant pattern. Case 8, originally presented with IVLBCL in the bone marrow and spleen, had a relapse in the lymph nodes with a DLBCL feature, where expression of CD8 was identified at both diagnosis and relapse. Case 27 was IVLBCL with a leukemic presentation and a similar weak expression pattern of CD4 was identified in the bone marrow and peripheral blood throughout. Case 11, initially diagnosed as FL without T-cell marker, transformed into CD8-positive DLBCL on relapse. In case 17, expression of CD8 was observed at diagnosis, but six months after complete response the disease relapsed as the same abdominal tumor without CD8 expression. Case 21 was originally considered a T-cell marker-negative DLBCL that relapsed in the testis with CD7 expression.

### Prognostic significance of T-cell marker expression in 169 patients with DLBCL

**Figure 3:**
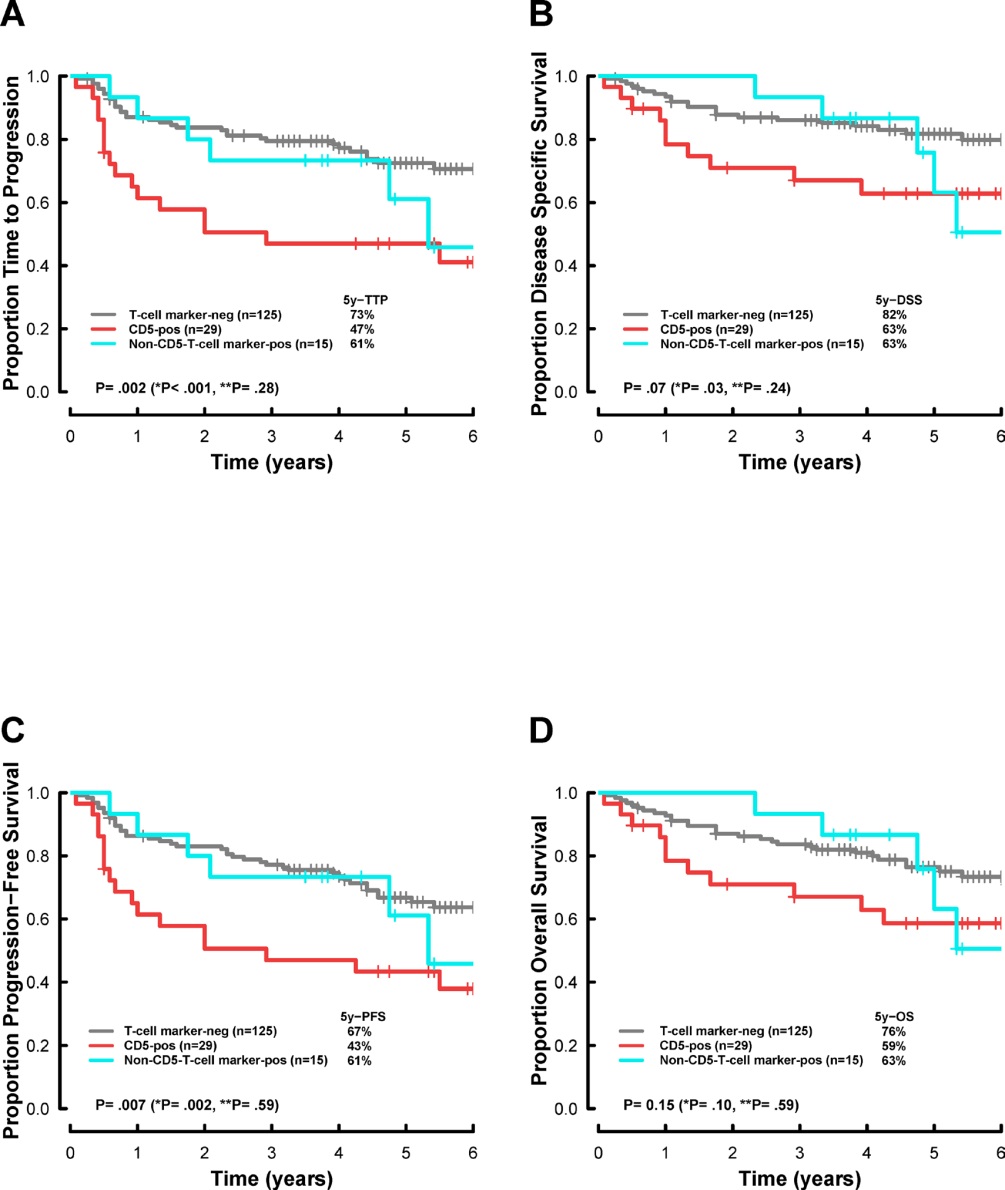
Survival analysis, according to T-cell marker status, in patients with newly diagnosed diffuse large B-cell lymphoma (DLBCL), treated with rituximab plus cyclophosphamide, doxorubicin, vincristine, and prednisone (R-CHOP)-based chemotherapy Kaplan-Meier curves represent **(A)** the time to progression (TTP), **(B)** disease-specific survival (DSS), **(C)** progression-free survival (PFS), and **(D)** overall survival (OS). T-cell marker-neg, T-cell marker-negative DLBCL; CD5-pos, CD5-positive DLBCL; Non-CD5-T-cell marker-pos, Non-CD5-T-cell marker-positive DLBCL. *P, CD5-pos vs. T-cell marker-neg; **P, Non-CD5-T-cell marker-pos vs. T-cell marker-neg. *P* values less than 0.05 were considered statistically significant.

The survival of patients with CD5-positive DLBCL was significantly poorer thanthat of patients with T-cell marker-negative DLBCL (Figures 3: 5-year time to progression (TTP), 47% vs. 73%, respectively, *p* < 0.001; 5-year disease specific survival (DSS), 63% vs. 82%, respectively, *p* = 0.03). In contrast, there was no significant difference in progression between non-CD5-T-cell marker-positive DLBCL and T-cell marker-negative DLBCL, suggesting that T-cell markers other than CD5 do not have prognostic impacts in DLBCL treated by immuno-chemotherapy (Figure 3).

Correlations between survival and basic parameters, including sex, International Prognostic Index (IPI), GC/non-GC phenotype, and T-cell markers (CD5, CD8, and CD7) are shown in Table 5. CD2 was not included in the univariate analysis because the number of CD2-positive patients was very small (n = 2). Multivariate Cox regression analysis was performed after excluding patients without T-cell marker expression because the patients in this group almost overlapped with CD5-negative patients. Univariate analysis showed that CD5 expression and high IPI were significant prognostic factors for TTP, progression-free survival (PFS), and DSS. Multivariate analysis revealed that high IPI remained a significant independent factor affecting TTP, PFS, DSS, and overall survival (OS). CD5 expression was significantly associated with shorter TTP (*p* = 0.01).

**Table 5:** Univariate and multivariate models using PFS and OS of 169 patients with DLBCL

variables	TTP			PFS			DSS			OS		
	HR	95% CI	p	HR	95% CI	p	HR	95% CI	p	HR	95% CI	p
Sex: male	0.77	0.45–1.30	0.32	0.78	0.48–1.26	0.31	1.04	0.54–1.98	0.91	1	0.57–1.78	0.99
IPI: 3–5	2.67	1.56–4.56	< 0.001	2.52	1.53–4.15	< 0.001	3.48	1.82–6.64	< 0.001	3.08	1.73–5.48	< 0.001
non-GC	1.77	1.04–3.01	0.035	1.56	0.96–2.54	0.072	2.02	1.05–3.87	0.035	1.63	0.92–2.90	0.093
CD5	2.69	1.52–4.78	0.001	2.31	1.34–3.99	0.003	2.08	1.01–4.31	0.048	1.7	0.87–3.35	0.12
CD8	1.5	0.64–3.49	0.35	1.25	0.54–2.89	0.61	0.61	0.15–2.54	0.5	0.48	0.12–2.00	0.32
CD7	1.82	0.66–5.05	0.25	1.49	0.54–4.12	0.44	1.01	0.24–4.22	0.98	0.76	0.19–3.15	0.71
Any T-cell marker*	2.37	1.39–4.05	0.002	1.94	1.17–3.22	0.01	2.07	1.08–4.00	0.03	1.57	0.86–2.88	0.14
**Multivariate**												
IPI: 3–5	2.54	1.48–4.37	0.001	2.44	1.45–4.02	< 0.001	3.33	1.74–6.38	< 0.001	3	1.68–5.35	< 0.001
non-GC	1.54	0.89–2.67	0.12	1.4	0.85–2.32	0.19	1.81	0.93–3.54	0.081	1.54	0.84–2.73	0.17
CD5	2.2	1.21–3.99	0.01	1.95	1.11–3.43	0.021	1.59	0.75–3.37	0.22	1.39	0.69–2.80	0.35

^*^ excluded from the multivariate analysis because this group referred to those who were negative for CD5. TTP, time to progression; PFS, progression-free survival; DSS, disease-specific survival; OS, overall survival; HR, hazard ratio; CI, confidence interval; non-GC, non-germinal center.

## DISCUSSION

The present study addressed how frequently T-cell markers were expressed in 501 B-cell lymphoma cases, diagnosed according to the 2008 WHO classification. In all cases, expression of T-cell marker was comprehensively investigated using FCM, and additional IHC analysis was successfully conducted for non-CD5-T-cell marker -positive cases. The expression of T-cell markers other than CD5, including CD8, CD7, CD2, and CD4, was identified in 27 patients, the largest number to date. Notably, all of these non-CD5-T-cell marker-positive cases were large B-cell lymphoma subtypes, which showed a significantly higher incidence than other common subtypes, such as FLs and MZLs. Studies evaluating expression of T-cell marker other than CD5 with a large sample size have been reported, but these results did not represent the demography of non-Hodgkin lymphoma subtypes. Carulli *et al* [13] reported eight patients with non-Hodgkin lymphoma showing aberrant expression of CD8 in 951 bone marrow samples, but that study was sharply biased in patient selection toward lymphomas with bone marrow invasion, such as CLLs/SLLs. In other studies [13, 17, 18] using more than 100 cases, variable histological classification, not the current WHO criteria, was applied, which does not represent the accurate distribution of non-Hodgkin lymphoma.

As for the prognostic relevance of T-cell marker expression, the present study found that only CD5 was a strong predictor of poor outcome in 169 patients with primary DLBCL, as previously reported [3]. Moreover, between non-CD5-T-cell-marker-positive and T-cell marker-negative DLBCLs, no significant difference in outcome was observed. To our knowledge, no survival data of T-cell marker-positive DLBCL has been presented in a large number of patients (>150 patients) uniformly treated with R-CHOP-based chemotherapy. Suzuki *et al* [31] were the first to investigate prognostic implications of T-cell markers other than CD5 in DLBCLs, and they showed that such expression was not associated with outcome, consistent with the present findings. However, in the study by Suzuki *et al*, [31] the survival data for only 92 patients treated with rituximab-based chemotherapy were available, and the frequency of T-cell marker other than CD5expression was only assessed in the DLBCL population. Additionally, in the survival analysis, only T-cell markers other than CD5 were evaluated, and co-expression of CD5 was not demonstrated. In our study, 5/29 patients with CD5-positive DLBCL simultaneously harbored additional T-cell markers other than CD5, although this redundancy appeared to have little influence on the disease outcome.

CD5-positive DLBCL has been reported, mainly from Japanese cohorts, to have unique clinical features. The present study shared common characteristics with previous studies with respect to unfavorable outcome, poor PS, non-GC subtype, extranodal disease, and female predominance. Compared with a large cohort study of Western populations by Xu-Monette *et al*., [32] the same clinical features as mentioned above were observed, but the present study found higher incidence (15% vs. 5.5%) of CD5 expression. This discrepancy may be attributed to difference in detection methods used (FCM or IHC) rather than the difference in genetic background. As described previously [33, 34], involvement of the central nervous system at the diagnosis or relapse was more frequently observed in CD5 positive cases (3/29; 10.3%) than in CD5 negative cases (5/140; 3.5%); however, this difference was not statistically significant (*p* = 0.11), probably owing to the small sample sizes.

As results of previous studies are based on the analysis of samples at diagnosis, the transition of T-cell marker expression has not been reported. We studied sequential samples of 7 patients at diagnosis and histologically-confirmed relapse/progression. Four patients presented identical expression of T-cell marker in the two sequential samples, indicating that both samples were clonally related and that the potential to express T-cell markers can be maintained even in different microenvironments. Of the remaining 3 patients, two displayed additional T-cell marker expression in the later samples (case 11, FL transformed; case 21, testicular relapse of DLBCL), probably due to additional genomic or epigenomic alterations. On the other hand, in case 17, expression of T-cell marker completely disappeared at relapse in the same biopsy site, suggesting that subclone(s) with additional alterations were specifically eradicated by chemotherapy, and other subclone(s) or stem line(s) without the alterations survived and predominated. Meanwhile, aberrant expression of T-cell markers is not necessarily a surrogate for the presence of subclones; phenotype plasticity has been well described and is the more probable cause of this phenomenon.

Although the expression of T-cell markers other than CD5 is uncommon in mature B-cell neoplasms, it has been described previously. The most frequently reported cases have been CD8^+^ CLLs, accounting for 0.5–3% of the total CLLs [6, 7, 10–13]. This frequency is consistent with the present finding that CD8 was the most frequently expressed marker among T-cell markers other than CD5, even though no CD8^+^ CLLs were found (0/13). The absence of CD8^+^ CLLs in the present study might be attributed to the infrequent incidence of CLL in Japan [35]. Other small B-cell lymphomas, such as FL, MZL, LPL, and MCL, express T-cell markers other than CD5 uncommonly [13, 17, 18], which is also consistent with our observations. As for large B-cell lymphomas, T-cell markers other than CD5 were expressed more frequently in immunocompromised patients, such as in lymphomas occurring in HIV-positive patients [14, 16], pyothorax-associated lymphomas [15, 19, 20], plasmablastic lymphomas [23, 24, 27, 29], and Epstein-Barr virus (EBV)-positive DLBCLs of the elderly [30], than in common DLBCL (Table 1). Most of these non-CD5-T-cell marker-positive large B-cell lymphomas were reported to express CD3 by IHC analysis. In contrast, no CD3-positive cases were observed in previous studies [9–13], or in the present study, where FCM was used for screening.

FCM has a higher sensitivity than IHC for detection of CD5 expression in DLBCL [3, 36]. Unlike other solid tumors, immunophenotyping of lymphomas is performed not only by IHC but also by FCM. In contrast to IHC, the expression of every representative lineage marker can be analyzed comprehensively by FCM, using a panel of antibodies. However, unlike IHC, which can be done at any time after biopsy, FCM can only be performed immediately after biopsy because it requires live unfixed cells. Therefore, the expression of T-cell markers other than CD5, such as CD2, CD4, CD7, and CD8, has not been broadly investigated in a large case series of B-cell lymphomas. Our FCM screening enabled a sensitive detection of T-cell marker-positive cases, and a good concordance between the FCM and IHC results supported the accuracy of FCM screening.

The human CD8 glycoprotein has two subunits, CD8α and CD8β, and the cell surface CD8 is assembled either as an αα homodimer or an αβ heterodimer, in the native state [37]. CD8αβ is broadly expressed on mature peripheral αβ T cells, whereas CD8αα is expressed on restricted subsets of cells, including γδ T cells, intestinal intraepithelial lymphocytes, natural killer cells, and dendritic cells [38, 39]. Rabinowitz *et al*. demonstrated that activation of T cells leads to the appearance of T-cell markers on the surface of neighboring B cells, through direct B- and T-cell interaction [40]. If this transfer mechanism had been operative for our cases, then each CD8^+^ neoplastic B cell in a lymphoma specimen would be positive not only for CD8α but also for CD8β, because most of the CD8^+^ T cells express both CD8α and CD8β, and they would probably have been transferred together, due to their robust disulfide-linked dimer structures. The majority of our CD8^+^ cases (15/18), however, were negative for CD8β (Table 2). This observation suggested that most of the CD8^+^ B-cell lymphoma cases harbored CD8αα not by antigen transfer, but rather by their own potential for abnormal gene expression. Our result was also consistent with a previous report that showed anomalous configuration of *CD8A* in a patient with CD8^+^ CLL, subsequently resulting in deregulation of *CD8B* gene expression [41].

In summary, our FCM-based study provided novel information about the proportion of non-CD5-T-cell marker-positive B-cell lymphomas, diagnosed according to the current WHO classification, and their prognostic significance in patients with DLBCL. CD5 was reconfirmed as a negative prognostic factor for DLBCL, whereas T-cell markers other than CD5 were shown to have no significance in clinicopathological and survival analyses. The mechanism of T-cell marker expression and the relationship between expression of CD5 and other T-cell markers remain unclear. However, these biological mechanisms should be further explored because of the reproducible prognostic importance of CD5-positivity in DLBCL.

## MATERIALS AND METHODS

### Case selection

We reviewed FCM data of cases that were consecutively diagnosed as mature B-cell lymphomas at the Cancer Institute Hospital, Japanese Foundation for Cancer Research (Tokyo, Japan), between March 2006 and June 2011. Five hundred and one newly diagnosed cases were retrieved, in which abnormal B-cell populations were detected by FCM and confirmed by histopathological examination. These 501 cases of mature B-cell lymphoma included 225 DLBCLs, 134 FLs, 81 MZLs, 17 MCLs, 13 CLLs/SLLs, 6 lymphoplasmacytic lymphomas, 4 Burkitt lymphomas, 2 IVLBCLs, 1 primary mediastinal large B-cell lymphoma, 1 DLBCL associated with chronic inflammation, and 19 low-grade B-cell lymphomas unclassifiable. The diagnostic criteria were based on the 2008 WHO classification.

Of the 225 DLBCL cases, 169 patients who received R-CHOP chemotherapy at our institution were selected to evaluate the prognostic impact of T-cell markers. Patients were excluded from survival analysis if they had a known prior history of an indolent lymphoproliferative disorder or a component of small B-cell lymphoma in the same biopsy specimen. There were no patients with HIV or testicular involvement at diagnosis. We obtained written informed consent from all patients included in the present study.

### Flow cytometry analysis

T-cell marker-positive B-cell lymphoma was defined when one or more T-cell markers (CD2, CD3, CD4, CD5, CD7, and/or CD8) were found by FCM, irrespective of the IHC results. Three-color flow cytometric immunophenotyping was performed, after selecting an appropriate lymphocyte gate on the combination of forward and side scatter plots. The antibodies used were kappa, lambda, CD2, CD3, CD4, CD5, CD7, CD8, CD10, CD11c, CD13, CD19, CD20, CD22, CD23, CD25, CD30, CD45, CD56, TCRαβ, and TCRγδ. The FCM dot plots were specially evaluated, through visual inspection, by two independent pathologists (N.T. and K.T.). Abnormal B-cell populations were determined by the expression of a single immunoglobulin light chain on CD19/CD20 positive cells, or the absence of both. We defined the staining results as follows: negative, similar to the intensity of negative controls (NC); positive, any degree of intensity greater than that of the NC. For T-cell markers determined as positive, we recorded details as follows; equally positive, similar to that of residual normal T cells; dimly positive, greater than that of NC and less than the residual normal T cell. When the distribution of dots extended from positive to negative, we explained the width of the distribution by using “to” as in equal to dim, dim to negative, and negative to dim.

### Histopathological analysis

Formalin-fixed, paraffin-embedded tissue sections were used for histopathological examination. Histopathological images were photographed using an Olympus BX51 microscope equipped with an Olympus UPlan SAPO 40x objective and a JVC KY-F75 digital camera. IHC was performed using a Dako Autostainer with the EnVision+ System-DAB(Dako, Glostup, Denmark), or a BOND-III with the Bond polymer Refine Detection kit (Leica Microsystems, Melbourne, Australia), with antibodies for CD5, CD10, CD20, BCL2, BCL6, MUM1, MYC, CD2, CD7, CD4, CD8, and CD8β (Table 6). DLBCL cases were classified into GC or non-GC phenotypes, according to the Hans algorithm [42]. For cases positive for non-CD5-T-cell markers by FCM analysis, additional immunostaining of T-cell markers was performed. On IHC analysis, cells were considered positive for T-cell markers when a small population of neoplastic cells was clearly positive. The cutoffs for MYC and BCL2 IHC were ≥ 40% and ≥ 50%, respectively. The presence of EBV was assessed by *in situ* hybridization with EBV-encoded small RNA. Fluorescence *in situ* hybridization (FISH) was performed on formalin-fixed, paraffin-embedded sections using three break-apart probes: *BCL2*, *BCL6*, and *MYC* FISH DNA split signal probes (Dako).

**Table 6:** List of antibodies used for immunohistochemistry

Antibody	Clone (Source)	Dilution
CD2	AB75 (Novocastra)	1:50
CD3	F7.2.38 (DAKO)	1:50
CD4	1F6 (Nichirei)	Ready to use
CD5	4C7 (Novocastra)	1:50
CD7	LP15 (Novocastra)	1:20
CD8	C8/144B(Nichirei)	Ready to use
CD8β	F-5 (SANTA CRUZ)	1:50
CD10	56C6 (Novocastra)	1:100
CD20	L26 (DAKO)	1:50
BCL2	124 (DAKO)	1:100
BCL6	PG-B6p (DAKO)	1:20
MUM1/IRF4	MUM1p (DAKO)	1:50
MYC	Y69 (Epitomics)	1:100

### Cytogenetic and gene rearrangement analysis

G-band karyotyping and gene rearrangement analysis were performed. For cases with availability of sufficient fresh biopsied material, *IgH* and *TCR* gene rearrangements were routinely analyzed by Southern blotting, using the JH and Cβ1 probes, respectively.

### Statistical analyses

For survival endpoints, the TTP, PFS, DSS, and OS were calculated from the date of diagnosis, according to Cheson's criteria [43]. The probabilities of TTP, PFS, DSS, and OS were calculated using the Kaplan-Meier method, and distributions were compared by the log-rank test. Univariate and multivariate analyses were performed with the Cox proportional hazard model. Data were analyzed using the R software package and Bioconductor (version 3.3).

## SUPPLEMENTARY MATERIALS TABLES


