# Supplementary material for: Clinical and prognostic significance of aberrant T-cell marker expression in 225 cases of de novo diffuse large B-cell lymphoma and 276 cases of other B-cell lymphomas
Source: Oncotarget. 2017 Mar 23;8(20):33487–500. doi: 10.18632/oncotarget.16532 (PMC5464884; doi:10.18632/oncotarget.16532)
Supplement: Supplementary file 1 [file oncotarget-08-33487-s001.pdf]

Clinical and prognostic significance of aberrant T-cell marker expression in 225 cases of *de novo* diffuse large B-cell lymphoma and 276 cases of other B-cell lymphomas

SUPPLEMENTARY MATERIALS

SUPPLEMENTARY TABLE

Supplementary Table: Cytogenetic findings of non-CD5-T-cell marker-positive DLBCLs with abnormal metaphases

Case No.	Karyotype
1	44,X,−Y,add(2)(p11),−3,add(3)(q27),−4,add(5)(p15),add(6)(q25),der(9)t(3;9)(q21;p24),add(11)(q11),−15,add(17)(p11),add(19)(p13),der(20)t(11;20)(q13;q11)ins(20;?)(q11;?),add(21)(p11),add(22)(q13),+mar1,+mar2[3]/42,idem,+add(4)(q21),−8,+add(11)(p11),−add(11),−13,−18,+20,−der(20)t(11;20)ins(20;?)[6]/46,XY[5]
4	47,X,−X,ins(1;?)(q21;?),add(2)(p11.2),add(2)(q11.2),+add(3)(q11.2),der(3;15)(q10;q10),add(6)(p21),add(7)(q32),−9,+10,+11,−13,add(13)(p11.2),add(17)(p11.2),+i(18)(q10),add(19)(q13.3),der(21)t(9;21)(q13;p11.2),+mar1[8]
6	46,X,−X,−1,del(6)(q?),−7,−15,−16,−17,−18,add(18)(q21),add(20)(q11.2),der(?)t(?;1)(?;q21),+r1,+mar1,+mar2,+mar3,+mar4,+mar5[16]
8	82,XXYY,add(1)(q42),der(1)add(1)(p11)add(1),−2,−3,?t(3;14)(q27;q32)x2,−4,−5,−6,−8,−8,add(10)(q22)x2,−11,−15,−16,del(16)(q?),−17,−19,add(19)(q13.1),−22,+mar1,+mar2,+mar3[3]/82,sl,add(11)(p11.2)[2]/46,XY[2]
9	54,XX,add(1)(p36.1),add(1)(q32),+2,add(2)(p11.2)x2,+add(3)(q27),add(11)(p11.2),+12,+12,−13,+5mar[1]/46,XX[1]
10	83,XXYY,−1,i(1)(q10),−3,add(3)(q27)x2,−6,−6,−8,−9,add(9)(p13),−12,del(13)(q12q14)x2,−14,−14,t(14;18)(q32;q21),−15,+16,−17,i(17)(q10)x2,−18,der(18)t(14;18),−19,−21,+r1x3,+mar[1]/46,XY[7]
11	45,XX,add(3)(p21),−4,−5,−6,add(8)(q13),add(10)(q22),−12,−13,−13,t(14;18)(q32;q21),add(17)(p11.2),mar1,mar2,mar3,mar4,mar5[20]
12	54,XY,+add(3)(q27),−5,del(6)(q?),add(7)(q22),−8,+16,+20,−22,+r,+7mar[1]
15	97,X,−X,−X,−Y,−Y,−2,add(2)(q21),−4,add(4)(q31),−5,−6,i(6)(p10),add(7)(q22)x2,−9,add(9)(p13),−11,−11,+12,−14,−15,−16,−17,−17,−17,−18,add(19)(q13.1),−21,+22mar[1]
16	48,XY,add(1)(q32),+add(3)(p21),add(3)(p21),add(6)(q13),add(7)(p13),der(9)add(9)(p13)inv(9)(p12q13),−13,−14,−15,−16,−17,+i(18)(q10)x2,−22,+mar1,+mar2,+mar3,+mar4[3]/46,XY,inv(9)(p12q13)[7]
21	47,XY,add(3)(q21),−5,add(8)(p11.2),del(12)(p?),?t(14;18)(q32;q21),add(17)(p11.2),−18,add(21)(p11.2),+3mar[1]
22	47,XY,t(3;22)(q27;q11.2),add(7)(q22),t(8;14)(q24;q32),del(9)(q?),add(10)(p11.2),add(19)(p13),+der(?)t(?;1)(?;q21)[8]/46,XY[1]
26	75,XX,+X,−Y,+1,add(2)(q31)x2,add(4)(q31),+5,−6,del(6)(q?),+11,+12,−13,−15,−15,−17,−18,+19,−22,+mar1,+mar2,+mar3,+mar4,+mar5,+mar6,+mar7,+mar8[2]/46,XY[13]
27	46,XX,add(1)(p32),add(5)(q35),−6,add(13)(p11.2),−14,add(14)(q32),+mar1,mar2[19]
